# Proof-of-Concept and Test-Retest Reliability Study of Psychological and Physiological Variables of the Mental Fatigue Paradigm

**DOI:** 10.3390/ijerph18189532

**Published:** 2021-09-10

**Authors:** Cayque Brietzke, Ítalo Vinícius, Paulo Estevão Franco-Alvarenga, Raul Canestri, Márcio Fagundes Goethel, Lucas Eduardo Rodrigues Santos, Bruno Viana, Tony Meireles Santos, Flávio Oliveira Pires

**Affiliations:** 1 Exercise Psychophysiology Research Group, School of Arts, Sciences and Humanities, University of São Paulo, São Paulo 03828-000, Brazil; cayquebbarreto@alumni.usp.br (C.B.); italovinicius@usp.br (Í.V.); francoalvarengape@gmail.com (P.E.F.-A.); raulcanestri@alumni.usp.br (R.C.); gbiomech@usp.br (M.F.G.); bferreiraviana1@yahoo.com.br (B.V.); tonymsantos@gmail.com (T.M.S.); 2 Human Movement Science and Rehabilitation Program, Federal University of São Paulo, Santos 11015-020, Brazil; 3 Physical Education, Estácio de Sá University UNESA, Resende 27515-010, Brazil; 4 Centre of Research, Education, Innovation, and Intervention in Sport, Faculty of Sport, University of Porto, 4200-450 Porto, Portugal; 5 Porto Biomechanics Laboratory (LABIOMEP), University of Porto, 4200-450 Porto, Portugal; 6 Graduate Program in Neuropsychiatry and Behavioral Sciences, Federal University of Pernambuco, Recife 50740-600, Brazil; lucas.rodriguessantos@ufpe.br

**Keywords:** mental exertion, neuropsychological test, psychophysiology, reproducibility

## Abstract

This study provided a proof-of-concept and test–retest reliability of measures frequently used to assess a mental fatigue paradigm. After familiarization, 28 healthy men performed (40-min) the Rapid Visual Information Processing (RVP) test in a test–retest design, having mental fatigue sensation, motivation, emotional arousal, total mood disturbance, and electroencephalography (EEG) in the prefrontal cortex measured before and after the test. EEG was recorded during a 3-min rest so that the power spectral density of theta (3–7 Hz) and alpha (8–13 Hz) bands was calculated. Pre-to-post RVP test changes in psychological and physiological domains were compared (paired-T tests), and absolute (standard error of measurement (SEM) and minimal difference (MD)) and relative reliability (intraclass correlation coefficient (ICC)) were calculated. The RVP test induced an increase (*p* < 0.05) in mental fatigue sensation (120.9% (109.4; 132.4)) and total mood disturbance (3.5% (−6.3; 13.3)), and a decrease in motivation (−7.1% (−9.2; −5.1)) and emotional arousal (−16.2% (−19.1; −13.2)). Likewise, EEG theta (59.1% (33.2; 85.0); *p* < 0.05), but not alpha band, increased due to RVP test. All psychophysiological responses showed poor-to-moderate relative reliability. Changes in mental fatigue sensation and motivation were higher than SEM and MD, but changes in EEG theta band were higher only than SEM. Mental fatigue sensation, motivation, and EEG theta band were sensitive to distinguish a mental fatigue paradigm despite true mental fatigue effects on theta activity may be trivial.

## 1. Introduction

Mental fatigue is a transitory state derived from high-demanding, self-sustained cognitive efforts, which is characterized by alterations in behavioral, psychological, and physiological domains [1,2]. From a behavioral perspective, mental fatigue may reduce performance and productivity in a number of cognitive and physical tasks, increasing the risk of mistakes and dangerous behavior in different areas such as military, sporting, industry, and services, as well as clinical and emergency attendance [3,4,5]. However, mental fatigue-derived repercussions in psychological and physiological domains have been less explored. In this regard, a comprehensive description of the mental fatigue state over these two dimensions may be important to plan strategies to reduce mental fatigue-derived errors in different workplaces. For example, the characterization of mental fatigue effects on psychological dimension may be important to validate useful and simple tools to be applied in workplaces. Moreover, the description of mental fatigue effects on physiological dimensions may help to unravel possible strategies to mitigate mental fatigue effects.

From a psychological perspective, mental fatigue is traditionally associated with an increased fatigue sensation described in terms of “tiredness” and “lack of energy” [1], but alterations in other perceptual responses such as motivation, emotional arousal, and mood have been more controversial [6,7,8,9]. For example, studies from exercise sciences fields showed that mentally fatigued individuals reported an increased fatigue sensation, although motivation was unchanged [6,10,11]. In contrast, others observed a reduced motivation and emotional arousal together with an increased mental fatigue sensation [7,9,12]. Accordingly, some showed changes in mood disturbance with mental fatigue [13,14,15], but others did not [6,16]. Although some methodological differences regarding the mental fatigue promotion may be related to these controversial results, there is a lack of studies investigating the reliability of psychometric instruments frequently used to assess mental fatigue effects on the psychological domain, thus the error of these instruments in a mental fatigue paradigm is not warranted. Evidence of reliability is important to assess the influence of the inherent error of measurement of validated tools [17,18] so that mental fatigue effects on the psychological domain measured through these variables require more investigation.

From the physiological domain, mental fatigue has been associated with several physiological variables [5,19,20,21,22]. Despite some studies suggesting that mental fatigue alters cardiovascular parameters such as heart rate variability and blood pressure [22,23], most relevant physiological alterations in mentally fatigued individuals include changes in cortical areas involved in top-down regulation [8,19,20,24,25]. An imaging study with fMRI observed a reduced activation and cerebral blood flow in frontoparietal cortical areas after a mental fatigue task [25]. Studies with electroencephalogram (EEG) have also demonstrated a slower activation in frontal and prefrontal cortical areas as indicated by an increased theta band power [7,8,9,20]. Recently, a meta-analysis confirmed the effects of mental fatigue on EEG theta and alpha band power in central, frontal, and posterior cortices in mentally fatigued individuals, thereby suggesting a relationship between mental fatigue and EEG slow frequency band oscillations [20]. The overall suggestion is that this slow-down activity in frontal and prefrontal cortical areas in mentally fatigued individuals is a consequence of a high-demanding and self-sustained cognitive task-induced adenosine accumulation in neuronal tissues [21].

Although studies have separately reported psychological and physiological responses to the mental fatigue paradigm [20,26,27], there is a paucity of studies showing a comprehensive description of mental fatigue effects on these dimensions. The use of different psychological variables together with a straightforward physiological response, rather than a single psychophysiological response in isolation, is necessary to provide a proof-of-concept of the mental fatigue effects on psychological and physiological dimensions. Furthermore, no study has yet investigated the reliability of variables frequently used to assess psychological and physiological dimensions in the mental fatigue paradigm, so that a proof-of-concept study of the mental fatigue effects should come together with reliability analysis of these measures. Somehow, controversial results regarding psychological and physiological dimensions in the mental fatigue paradigm may be related to the lack of a straightforward methodology to measure mental fatigue [6,7,28,29,30,31].

This study aimed to provide a proof-of-concept of the mental fatigue effects on psychological and physiological domains, together with reliability analysis of variables frequently used to assess these dimensions. Separate evidence from different fields [32,33] may support the hypothesis that psychological variables, such as mental fatigue sensation, motivation, emotional arousal, and mood, as well as physiological variables such as EEG theta and alpha band measured in the prefrontal cortex, would show good sensibility and reliability to detect pre-to-post changes in healthy participants submitted to a high-demanding, self-sustained cognitive task.

## 2. Materials and Methods

### 2.1. Design and Participants

Importantly, a total sample size of 28 participants would provide a power of analysis higher than 0.82, considering a moderate mental fatigue effect size (f = 0.25) and a 5% alpha error in pre-to-post mental fatigue test measures. Therefore, this proof-of-concept (phase I) and test–retest reliability (phase II) study was carried out within four visits with 28 healthy men recruited from an announcement in social media (i.e., Facebook and Instagram). We recruited only participants falling into the inclusion criteria such as being healthy men (without known or apparent illness), free from cognitive and brain disorders that could potentially limit the comprehension of the psychological scales or disrupt the cognitive task test execution. Participants performed a first familiarization with mental fatigue test and psychological scales after assessment of age and anthropometric data. In the second visit, they repeated all the familiarization procedures used in the first visit. In the third visit, participants performed a high-demanding, self-sustained cognitive task having several psychophysiological measures assessed before and immediately after the mental fatigue test. In the fourth visit, 19 out of the 28 participants performed an extra mental fatigue test for reliability proposals. The visits were separated by a 3–7-day period, and participants were asked to maintain the individual diet schedule and avoid exhaustive physical exercises for the 48 h preceding the visits. This design, consisting of two familiarization visits before the experimental ones, served to reduce intra-individual variability as much as possible and ensure a suitable ICC analysis. All participants signed the consent term after an explanation of the risks and benefits of the study. This study was approved by the local Ethics Committee (54910716.4.0000.5390) and conformed to the Declaration of Helsinki. All procedures and results were reported according to the Guidelines for Reporting Reliability and Agreement Studies (GRRAS) [34].

### 2.2. Mental Fatigue Protocol

Mental fatigue was induced through the Rapid Visual Information Processing test (RVP), as suggested elsewhere [7,8,9,35,36]. The RVP is a high-demanding, sustained attention and inhibitory control cognitive task known to induce changes in cerebral and psychological variables when performed for a period longer than 20 min [7,35,36]. Then, to increase the likelihood of installing mental fatigue, participants performed a 40 min RVP test. They seated in a comfortable chair in front of a 17-inch monitor, wearing earplugs to avoid environmental noises. The test was conducted in a quiet and well-illuminated room so that participants could sustain attention on the task progression without distraction. The RVP test consists of identifying sequential three even (i.e., 2, 6, 4) or odd (i.e., 3, 5, 9) numbers randomly presented on the screen in a frequency of one number per 600 ms. Participants pressed the spacebar of the keyboard when they identified the correct sequential numeric order. An experienced researcher continuously monitored the RVP test to ensure that participants were totally engaged in pursuing the best performance. The researcher always verbally asked for engagement when participants dismissed three correct number sequences. The number of correct answers (times) and false alarms (times), as well as the reaction time (s), were reported and interpreted as measures of cognitive performance [8,9,37].

### 2.3. Psychological and EEG Measurements

Psychophysiological measures that have been frequently assessed separately in mental fatigue studies were included in the present study. To assess the psychological domain of the mental fatigue paradigm, we assessed a number of perceptual responses such as mental fatigue sensation, motivation, arousal, and mood disturbance. Mental fatigue sensation was obtained through a visual analog scale (VAS) ranging from 0 to 100 mm, anchored as “0” (zero) and “100” when participants reported ‘‘none at all’’ and ‘‘maximal’’ mental fatigue sensation, respectively [38]. Participants were asked to exclude perceptions of physical fatigue when rating mental fatigue sensation by answering the question “how mentally fatigued do you feel now?” Motivation was assessed through an 11-point Likert scale (0–10) having two contrasting descriptors such as “not all motivated” and “extremely motivated”, referenced to “0” (zero) and “10”, respectively. Intermediate qualitative descriptors were weakly, moderately, and strongly motivated, referenced to “2”, “5” and “8” in the motivation scale, respectively. Participants were asked to rate their motivation when answering the question “how motivated to do a physical test you feel now?” We assumed that a scale questioning the willingness to do a physical effort after a high-demanding cognitive task would best reflect how mental fatigue affects motivation. Emotional arousal was assessed through a 6-point Likert scale (Felt Arousal Scale) ranging from “low arousal” to “high arousal”. Participants were oriented to answer the question “how worked-up you feel?” when rating a “low arousal” as “1” (i.e., sensation of relaxation or calmness) or “high arousal” as “6” (i.e., sensation of worked-up or anxiety) [39]. Mood was obtained through the Profile of Mood States (POMS) questionnaire, consisting of 42 indicators with six domains of humor [40]. We calculated the total mood disorder as the sum of different mood domains such as anger, confusion, depression, fatigue, and tension, subtracted from the vigor domain and summed by 100. Measures of mental fatigue sensation, motivation, emotional arousal, and mood were obtained before and immediately after the RVP test conclusion in random order.

The physiological domain of the mental fatigue paradigm was obtained by EEG responses recorded in the prefrontal cortex. Briefly, we decided to measure the prefrontal cortex activity due to its role in inhibitory control and sustained attention [41]. The EEG signal was sampled through an EEG digital unit (Emsa^®^, EEG BNT 36, TiEEG, Rio de Janeiro, Brazil), having active Ag-AgCl electrodes placed at the Fp1 position, according to the EEG international 10–20 system. The electrode placement was oriented by the nasion and inion positions and the signal recorded was referenced to the mastoid process [42]. After cleaning and exfoliating the electrodes’ areas, we applied a conductive gel and fixed electrodes with medical straps. The EEG was sampled in a turned-off lightroom during a 3 min period before and immediately after the RVP test (immediately before participants rate their perceptual responses), while participants were resting with eyes closed. The EEG signal was captured with a 600 Hz frequency and Notch filter (60 Hz); thereafter the EEG data were filtered with a 3–50 Hz band-pass recursive filter. The initial 30 s time window was removed from the pre-test and post-test record dataset to avoid eventual noise associated with the individuals’ expectation of initiating the EEG record; then the most steady signal within 5 s time windows of the remaining EEG signal was processed [8,9]. The power spectral density (PSD) of the EEG theta (3–7 Hz) and alpha (8–23 Hz) band frequencies was calculated throughout a Fast Fourier Transform algorithm so that an increase in these low-frequency bands was interpreted as a mental fatigue-induced response, as suggested elsewhere [20]. All EEG data were processed and analyzed with specially programmed routines within the Matlab environment (Version 8.5.0.197613; MathWorks, Inc., Natick, MA, USA).

### 2.4. Statistical Analysis

In the proof-of-concept analysis, pre-to-post RVP test changes in psychophysiological variables such as mental fatigue sensation, motivation, emotional arousal, total mood disorder, fatigue sensation, and EEG PSD were obtained with a paired student-T test. The behavior domain of the mental fatigue paradigm was assessed as reaction time, correct answers, and incorrect answers, thus we compared how these responses progressed during the RVP test through a linear mixed model having a moment as a fixed factor (i.e., 25%, 50%, 75%, and 100% of the test) and participants as a random factor.

Reliability analysis was performed and interpreted according to standard recommendations [43]. Briefly, intraclass correlation coefficient (ICC) is a relative reliability index that allows assessing the relationship between scores from two trials. Therefore, we used a two-way mixed-effects ICC (ICC_(3,1)_) as shown in Equation (1), reporting it as confidence interval 95% (CI 95%) and significance at *p* < 0.05. Thereafter, the ICC_(3,1)_ was interpreted as having poor (<0.5), moderate (≥0.5 and <0.75), good (≥0.75 and <0.90) and excellent reliability (>0.90), as suggested elsewhere [43].

(1) $${\mathrm{ICC}}_{\left(3,1\right)}=\frac{{\mathrm{MS}}_{R}-{\mathrm{MS}}_{E}}{{\mathrm{MS}}_{R}+\left(k-1\right){\mathrm{MS}}_{E}}$$

where MS_R_ is the mean square for rows, MS_E_ is the mean square for error, and k is the number of measurements [43].

Furthermore, psychological responses from Likert scales were rescaled to a 100-point amplitude when necessary. For example, emotional arousal responses were redimensioned from 0–6-point to 0–100-point. Accordingly, EEG responses were rescaled to 0–100-point, having the lowest EEG value considered as “0” (zero) and the highest as “100”. This rescaling procedure was used to ensure proportionality across variables of different scales, thereby allowing the absolute reliability to be reported as a percentage of alteration in the standard error of measurement (SEM) and minimal difference (MD) [44]. This approach provided a fair comparison of variability from different scales-derived scores between trials. Both SEM and MD were calculated as shown in Equations (2) and (3), being interpreted in combination with the percentage of pre-to-post RVP test change [45].

(2) $$\mathrm{SEM}=\sqrt{\mathrm{MSE}}$$

where SEM is the standard error of measurement

(3) $$\mathrm{MD}=\mathrm{SEM}\times 1.96\times \sqrt{k}$$

where MD is the minimal difference and k is the number of trials.

Results were reported as mean and standard deviation (±SD) and 95% CI. We further calculated the effect size as Cohens’ d (“d”) for each comparison. Analyses were carried out in Rstudio software (v 1.3.1093) using native functions, pwr, and psych packages.

## 3. Results

### 3.1. Proof-of-Concept of the Mental Fatigue

Results demonstrated that mental fatigue altered psychological domain responses, as mental fatigue sensation (*p* < 0.001; d = 1.09) and total mood disturbance (*p* = 0.04; d = 0.43) increased due to the RVP test execution. In contrast, participants reported a decrease in motivation (*p* = 0.02; d = 0.19) and emotional arousal (*p* = 0.001; d = 0.42). Regarding the physiological domain, paired comparisons showed that RVP test execution increased EEG theta band (*p* < 0.001; d = 0.47), but not EEG alpha band (*p* = 0.055; d = 0.29). Results of both psychological and physiological domains are reported in Table 1.

Alterations in psychological and physiological domains were further accompanied by alterations in behavioral domain, as reaction time significantly decreased during the RVP test (*p* = 0.049; d = 0.51). However, neither the number of correct answer (*p* = 0.148; d = 0.40) nor incorrect answers (*p* = 0.963; d = 0.29) significantly changed as RVP test progressed.

### 3.2. Reliability of Mental Fatigue Related-Variables

Relative reliability analysis as measured by ICC_(3,1)_ showed poor-to-moderate reliability in all psychophysiological measures. For example, mental fatigue sensation (*p* = 0.01, ICC_(3,1)_ = 0.50) and total mood disturbance (*p* = 0.003, ICC_(3,1)_ = 0.59) showed moderate reliability, whilst motivation (*p* > 0.05, ICC_(3,1)_ = 0.21) and emotional arousal showed a poor and non-significant relative reliability (*p* > 0.05, ICC_(3,1)_ = 0.36). Furthermore, we observed a poor and non-significant relative reliability either in EEG theta band (*p* > 0.05, ICC_(3,1)_ = 0.32) or in EEG alpha band (*p* > 0.05, ICC_(3,1)_ = 0.30).

In contrast, absolute reliability combined with pre-to-post RVP change revealed consistent reliability for measures derived from VAS, motivation scale, and EEG theta band. For example, mental fatigue sensation due to the RVP test execution changed 120.9%, being highly above both SEM (26.1%) and MD (72.3%). Accordingly, motivation changed −7.1% from the pre-to-post RVP test, being above SEM (1.5%) and MD (4.2%). Regarding EEG results, the 59.1% change in theta band was just at the MD (59.1%), falling well above the calculated SEM (21.3%), perhaps suggesting a true, but trivial mental fatigue effect in the EEG theta band. Neither the remaining psychological responses, such as emotional arousal and total mood disturbance, nor EEG alpha band showed a pre-to-post RVP test change higher than SEM so that the absolute reliability of these variables is not warranted. Table 2 shows relative and absolute reliability results of psychophysiological variables.

## 4. Discussion

This study provided a proof-of-concept and test–retest analysis of psychophysiological variables frequently used to assess the mental fatigue paradigm. Overall, results showed that VAS and MOT were sensible and superior to correctly detect mental fatigue-derived alterations in the psychological dimension. Accordingly, the EEG theta band measured in the prefrontal cortex was sensible and superior to detect a mental fatigue state in the physiological dimension.

We observed that psychophysiological responses assessed through VAS, motivation scale, and EEG theta band were sensible to identify a mental fatigue paradigm induced by 40 min of a high-demanding cognitive task, as responses derived from these tools showed a significant pre-to-post change (from 7.1% to 120.9%) which was superior to the associated SEM. The fact that the pre-to-post change and SEM in mental fatigue sensation and motivation responses were 120.9% vs. 26.1% and −7.1% vs. 1.5%, respectively, may be interpreted as low chances of having a false-positive error when assuming mental fatigue through these variables. In contrast, despite being significant, the pre-to-post RVP test change in total mood disturbance and emotional arousal fell within the SEM calculated for POMS and Felt Arousal Scale, thus suggesting a false positive outcome if assuming mental fatigue through these tools. Likewise, theta, rather than alpha, activity was sensible in detecting a mental fatigue paradigm with EEG measures, given the change and SEM in theta and alpha band, 59.1% vs. 21.3% and 28.5% vs. 35.1%, respectively. Indeed, EEG studies have suggested that the EEG theta band is preferable to distinguish a mental fatigue state [5,19,20]. Hence, previous studies had little chances of error if assuming mental fatigue based on one of these responses [7,14,19,20,46].

Prior studies have reported controversial results in variables frequently used to assess a mental fatigue paradigm in psychological [6,7,9,10,11,12] and physiological domains [20,47,48], perhaps due to the error and variability associated with these tools, therefore highlighting the importance of reliability analysis of these responses. Indeed, such controversy may have been related to some “false positive” results when assessing mental fatigue through these tools. Somehow, the poor relative reliability of these variables as indicated by ICC results may be involved in such a controversy, as the error inherently present on these tools may have influenced the scores obtained with some of them. However, this is not necessarily a contradiction with the suggestion of superiority of VAS and motivation scale in detecting a mental fatigue state in the psychological domain, neither EEG theta band being superior in the physiological domain, as the suggestion of superiority of these variables was based on absolute rather than relative reliability. We discuss these reliability indexes to provide directions for future practical and theoretical studies on the mental fatigue paradigm.

Analysis of relative reliability is important to set the variability of a given measure when performed repeatedly, as this assesses the influence of the inherent error of measurement on the results [17,34]. The ICC is a measure of relative reliability, indicating the ratio between individual values variance and total variance in test–retest so that ICC is highly affected by the within-subject variability. As a result, ICC is highly affected by variations in the psychophysiological status of participants and experimental procedures. In the present study, we took the precaution to reduce the participants’ variability between sessions, familiarizing them with psychometric and electrophysiological instruments in two visits and recommending them to maintain the individual diet schedule and avoid exhaustive physical exercise for the 48-h preceding the procedures. However, the infeasibility in providing a more comprehensive control over daily activities that directly affect these psychological and EEG measures may challenge the relative reliability of these variables, as a comprehensive control would also require participants to avoid social interactions, work, and study routines, etc. For example, despite the efficacy in installing mental fatigue with the RVP test in most participants, some presented a high between-trial variability on mental fatigue sensation scores, suggesting that the psychological status at the pre-test condition was highly variable. Accordingly, despite using rigorous procedures when preparing participants for EEG recordings, we observed high variability in the EEG signal. Therefore, a challenge in mental fatigue paradigm studies is to provide a comprehensive and feasible control to ensure as low a within-subject variability as possible on psychophysiological status when using psychometric and EEG measures.

Despite being a useful relative reliability index for controlled settings in scientific investigations, ICC has limited applicability to inform the reliability of a given measure in real-world scenarios such as sports, labor, and military settings, or clinical and emergency services [44,49]. In this sense, absolute reliability, as indicated by SEM and MD calculation, likely provides a more practical index of reliability, as they can assess the error of measurement through values that maintain properties of the original tools, being expressed as original or rescaled units (i.e., relative to a maximal value). Consequently, these indexes allow setting a real, clinically relevant difference between two evaluations. For example, we found a 26.1% SEM in VAS results, indicating that individuals rating a mental fatigue sensation above this score threshold had a true mental fatigue effect. The fact that we also found a pre-to-post RVP test changes higher than MD (i.e., 120.9% vs. 72.3%) reinforces the high degree of certainty if assuming a mental fatigue state through VAS. Likewise, pre-to-post RVP test changes in motivation were also higher than MD, then further suggesting a high degree of certainty if assuming mental fatigue state through motivation scale. Thus, future studies are recommended to consider these results if aiming to distinguish a mental fatigue paradigm with a fair level of certainty.

Furthermore, RVP test-derived changes in EEG theta band were just at the calculated MD, that is 59.1% vs. 59.1%, respectively. The fact that significant changes in EEG theta band were higher than SEM but just at the MD may suggest a true, but trivial, mental fatigue effect on theta activity measured at Fp1 position (prefrontal cortex). A recent systematic review and meta-analysis [20] found a large mental fatigue-induced increase in EEG theta band measured in the frontal cortex so that the authors suggested the use of theta activity as a robust biomarker of mental fatigue. These results do not necessarily contradict our reliability results, as meta-analysis considers the effect of a given intervention without considering the error of measurement associated with that intervention. Moreover, methodological differences involving the type and duration of the cognitive task used to induce mental fatigue as well as EEG procedures should be considered. Together with the review study [20], our results show that the EEG theta band is a sensible variable to distinguish a real mental fatigue state, despite that one may argue that changes may be trivial rather than large in some experimental settings. Future studies and clinical interventions aiming at using EEG theta band in the mental fatigue paradigm should make efforts to standardize procedures to increase the likelihood of observing true mental fatigue effects.

### Methodological and Practical Applications

Some points should be considered in future mental fatigue paradigm studies. Firstly, proof-of-concept as well as absolute reliability results indicated that VAS, motivation scale, and EEG theta band were sensible to distinguish a mental fatigue paradigm. These results have applications to controlled settings as well as real-world contexts, as mental fatigue researchers have good chances to make correct decisions when assuming mental fatigue state through these responses. Given the practicability and low-cost aspects of psychological scales, mental fatigue sensation and motivation may be preferable in most real-world scenarios. However, one may argue that VAS is preferable than motivation, as VAS likely best fits to the mental fatigue definition of “an increased fatigue sensation described in terms of “tiredness” and “lack of energy” [1].

Secondly, despite familiarizing participants with scales and the RVP test in two preliminary visits and conducting measures with rigorous control, we observed a reasonable variability in mental fatigue state as rated by VAS at baseline (pre-intervention with RVP test). Given that mental fatigue is possibly associated with a variety of different daily activities including traffic, diet restrictions, work and household routines, scholar tests, and others, participants may have arrived at the laboratory eliciting a varied level of mental overload. Somehow, such a varied mental fatigue state at baseline can have contributed to a reduced reliability level observed in some instruments. Future mental fatigue studies should plan strategies to control possible sources of mental overload, thus reducing the variability of the participants’ mental state before testing. For example, studies may recommend participants to avoid sleep deprivation and cognitive and scholar tests during the hours preceding experimental mental fatigue trials to obtain adequate pre-intervention control. In addition, researchers of the mental fatigue paradigm may plan washout strategies to standardize the participants’ mental state at the arrival in the laboratory. For example, short resting periods (10–15 min) while listening to neutral music such as meditation songs may have the potential to recover the participants’ mental state before the mental fatigue intervention.

Thirdly, in the present study we studied mental fatigue-related responses by using a specific high-demanding self-sustained cognitive task, the RVP test. However, one may want to investigate psychological and physiological domains when mental fatigue is induced by other cognitive tests such as Stroop Test, AX-CPT, and others [6,8,35,50]. Although future studies are necessary to confirm these results when mental fatigue is induced by other cognitive tests and strategies, we have no reasons to suspect that such results would be extraordinarily different from those reported here, if considering comparable cognitive workloads. Therefore, we believe that our absolute reliability results are somehow transferable to real-world scenarios in which the easy mental fatigue assessment is important to prevent risks of accidents such as in ground and air transportation, military maneuvers, or emergency medical services.

To the best of our knowledge, there is no gender-related difference in mental fatigue paradigm as both men and women may be equally subjected to mental fatigue. In the present study, the inclusion of healthy men was a methodological strategy to ensure that participants would be capable to accomplish the research protocol with RVP test. Hence, we have no reason to avoid inferences to adults of both genders. However, we acknowledge that the cognitive age may be a factor potentially affecting the mental fatigue paradigm, so that future studies are required to confirm these results in younger and elderly people.

## 5. Conclusions

Results of the present study showed that VAS, motivation scale, and EEG theta band were sensible to assess the mental fatigue paradigm in healthy men. While changes in EEG theta activity may be trivial, VAS and motivation seem to be highly useful and practical to distinguish mental fatigue state in both laboratory and real-world scenarios.

## Figures and Tables

**Table 1:** Psychological and physiological variables frequently used to assess mental fatigue.

Measure	Pre-RVP	Post-RVP	Change (%)	*p*-Value	ES
Mental fatigue sensation (a.u.)	23.3 ± 21.8 (14.8; 31.8)	51.4 ± 28.6 (40.4; 62.5)	120.9 ± 31.0 (109.4; 132.4)	<0.001	1.09
Motivation (a.u.)	7.0 ± 2.5 (6.0; 8.0)	6.5 ± 2.7 (5.5; 7.5)	−7.1 ± 5.4 (−9.2; −5.1)	0.020	0.19
Emotional arousal (a.u.)	6.0 ± 2.2 (5.1; 6.8)	5.0 ± 2.3 (4.1; 5.9)	−16.2 ± 8.0 (−19.1; −13.2)	0.001	0.42
Total mood disturbance (a.u.)	101.5 ± 10.3 (97.6; 105.5)	105.1 ± 13.0 (100.1; 110.1)	3.5 ± 26.5 (−6.3; 13.3)	0.038	0.43
EEG theta band (dB/Hz)	55.2 ± 39.8 (40.5; 71.4)	89.0 ± 67.7 (62.7; 115.2)	59.1 ± 69.9 (33.2; 85.0)	<0.001	0.47
EEG alpha band (dB/Hz)	130.3 ± 135.2 (77.9; 182.7)	167.4 ± 117.2 (122.0; 212.9)	28.5 ± 13.3 (23.6; 33.4)	0.055	0.29

Values are the mean ± SD (CI95%); CI95% is 95% confidence interval; RVP is the rapid visual information processing (the mental fatigue test); ES is effect size (d = tc2 (1 − r) n); EEG is electroencephalography; and EEG measured at Fp1 (prefrontal cortex).

**Table 2:** Relative and absolute reliability analysis of psychological and physiological variables frequently used to assess mental fatigue.

Measure	ICC_(3,1)_ (IC_95%_)	ICC_(3,1)_ (IC_95%_) General Guide ^2^	*p*-Value	SEM (%)	MD (%)
Mental fatigue sensation ^1^ (a.u.)	0.50 (0.15; 0.74)	Moderate (poor; moderate)	0.012	26.1	72.3
Motivation (a.u.)	0.21 (−0.19; 0.54)	Poor (poor; moderate)	0.19	1.5	4.2
Emotional arousal (a.u.)	0.36 (−0.02; 0.65)	Poor (poor; moderate)	0.059	21.9	60.6
Total mood disturbance (a.u.)	0.59 (0.27; 0.79)	Moderate (poor; good)	0.003	6.2	17.3
EEG theta band (dB/Hz)	0.32 (−0.07; 0.62)	Poor (poor; moderate)	0.086	21.3	59.1
EEG alpha band (dB/Hz)	0.30 (−0.09; 0.61)	Poor (poor; moderate)	0.10	35.1	97.1

95% CI is 95% confidence interval; ICC is intraclass correlation; SEM is standard error of measurement; and MD is minimal difference; Reliability results were expressed as a percentage of original or rescaled results (0–100%); ^1^ Mental fatigue sensation assessed through VAS; ^2^ Koo, T. K., & Li, M. Y. (2016). A Guideline of Selecting and Reporting Intraclass Correlation Coefficients for Reliability Research. Journal of chiropractic medicine, 15(2), 155–163. https://doi.org/10.1016/j.jcm.2016.02.012, accessed on 12 October 2020.

## Data Availability

The data are available upon requested from corresponding author.
